# Opportunistic hand radiographs to screen for low forearm bone mineral density: a prospective and retrospective cohort study

**DOI:** 10.1186/s12891-023-07127-w

**Published:** 2024-02-20

**Authors:** Alana O’Mara, Faes Kerkhof, Deborah Kenney, Nicole Segovia, Paige Asbell, Amy L. Ladd

**Affiliations:** 1 Department of Orthopaedic Surgery, Stanford University, Stanford, CA USA; 2 Department of Orthopaedic Surgery, Robert A. Chase Hand & Upper Limb Center, Stanford University, Stanford, CA USA

**Keywords:** Bone mineral density, Forearm fractures, Osteoporosis, Hand x-rays

## Abstract

**Background:**

Low bone mineral density affects 53% of women over age 65 in the US, yet many are unaware and remain untreated. Underdiagnosis of forearm osteoporosis and related fragility fractures represent missed warning signs of more deadly, future fractures. This study aimed to determine if hand radiographs could serve as early, simple screening tools for predicting low forearm bone mineral density (BMD).

**Methods:**

We evaluated posterior-anterior (PA) hand radiographs (x-rays) and Dual-energy X-ray absorptiometry (DXA) scans of 43 participants. The ratio of the intramedullary cavity to total cortical diameter of the second metacarpal (second metacarpal cortical percentage (2MCP)) was used as a potential diagnostic marker. Mixed-effects linear regression was performed to determine correlation of 2MCP with BMD from various anatomic regions. Repeated measures ANOVAs were used to compare BMD across sites. An optimal 2MCP cutoff for predicting forearm osteopenia and osteoporosis was found using Receiver Operating Curves.

**Results:**

2MCP is directly correlated with BMD in the forearm. The optimal 2MCP of 48.3% had 80% sensitivity for detecting osteoporosis of the 1/3 distal forearm. An 2MCP cutoff of 50.8% had 84% sensitivity to detect osteoporosis of the most distal forearm. Both 2MCP cutoffs were more sensitive at predicting forearm osteoporosis than femoral neck T-scores.

**Conclusions:**

These findings support the expansion of osteoporosis screening to include low-cost hand x-rays, aiming to increase diagnosis and treatment of low forearm BMD and fractures. Proposed next steps include confirming the optimal 2MCP cutoff at scale and integrating automatic 2MCP measurements into PAC systems.

**Supplementary Information:**

The online version contains supplementary material available at 10.1186/s12891-023-07127-w.

## Background

Low bone mineral density (BMD) afflicts 53% of the women over the age of 65 in the US and is expected to increase as the population ages [1]. However, many are still unaware of their condition and remain untreated [1–4]. This leads to an appreciable risk of fragility fracture, which is a fracture caused by a low-energy mechanism [5]. In 2005 alone, osteoporosis accounted for 2 million new fractures and is predicted to rise by almost 50%, underscoring the need to address low BMD before fractures occur [6, 7].

Specifically, fragility fractures of the forearm have been considered strong indicators of subsequent fractures [8]. Cuddihy demonstrated that after sustaining a distal radius fracture, there is a 1.4 fold increase in hip fractures – which have a one-year mortality rate of 16–21% if surgically addressed [8–13]. Furthermore, even after correcting for hip fractures, BMI, smoking and other comorbidities, osteoporosis of the distal forearm is related to a 1.32 fold increased risk of mortality in women, highlighting the potential value of assessing forearm BMD [10]. This suggests low forearm BMD is an independent risk factor for developing future fractures and mortality.

However, screening for low BMD of the forearm is limited. The gold standard for screening is a dual-energy x-ray absorptiometry (DXA) which measures BMD at various anatomical regions [5]. Fracture risk is then calculated using the FRAX tool, which utilizes BMD solely from the weight-bearing femoral neck region [14, 15]. Consequently, these estimates do not accurately reflect fracture risk in the non-weight-bearing forearm [14].

Additionally, there has been a reduction in DXA screening after Medicare reimbursement cuts in 2006 leaving many patients with osteoporosis unscreened and untreated [16]. Previous studies showed that 74% of Medicare beneficiaries who sustained a distal radius fracture were never tested for osteoporosis with DXA scans within 2-years, and only 7% were tested within 6 months of fracture [17]. In contrast, those who were tested had longer intervals to second fracture compared to patients without testing (819 vs. 579 days) [17]. A similar investigation on fragility fractures of the radius revealed up to 70% of patients had osteoporosis at the time of injury but lacked a formal diagnosis, 19.3% then went on to sustain a second fracture, and none were referred to an endocrinologist for preventive treatment at the time of discharge [3].

These studies demonstrate how frequently osteoporosis is missed or undertreated, especially in the forearm. Hand radiographs (x-rays) were likely accessible for all the patients with forearm fractures and constitute a missed opportunity for osteoporosis screening. This is because several studies have shown x-rays correlate to BMD by quantifying cortical thickness [18–20]. Pioneering work by radiologists in the 1960s and 70s, used the PA view of hand x-rays to measure cortical thickness as a new approach to diagnosis osteoporosis; their main limitation at the time was the small scale for measurement which has now been obviated by using computer processing systems. Early on, these physicians demonstrated high inter-observer agreement, a high degree of correlation with cortical bone mass, and ease of use [21–23]. Hand x-rays capture the cylindrical cortices of the metacarpals which can be utilized to evaluate cortical thinning at various angles [24]. Furthermore, hand and wrist x-rays are routinely ordered in many primary care, orthopaedic, and hand practices. As a result, hand and wrist x-rays represent a cost-effective, important opportunity for further screening and/or intervention for forearm osteopenia and osteoporosis.

In juxtaposition to the aforementioned grim osteoporosis undertreatment and non-diagnosis statistics, early screening and treatment for osteoporosis has shown to be remarkably beneficial. Research shows a 25.9% reduction in fracture risk at any site for those screened before the standard age of 65 [25, 26]. Screening specifically for forearm BMD is extremely beneficial as it has a much stronger odds ratio (3.98) to predict distal radius fractures than BMD from the hip (OR = 0.27) or femoral neck (OR = 0.26) [27]. Expanded screening tools could help patients receive calcium supplementation and or vitamin D, start on osteoporosis treatment, forestall further injury, or disability.

Therefore, opportunistic hand and wrist x-rays could provide physicians with an early screening tool that is more feasible, cost-effective, and considers non-weight-bearing regions compared to standard DXAs to determine osteopenia or osteoporosis risk.

The purpose of this study was to determine if cortical bone thickness measured from hand x-rays correlated with BMD in the forearm. We chose to study only females as the incidence of low bone mineral density and associated fragility fractures is higher .We hypothesized that the 2MCP would be (1) directly related to DXA scores from the radius, (2) be non-inferior to BMD as determined by DXA, and (3) is a superior estimator of osteopenia and osteoporosis of the forearm compared to the current estimates generated from weight-bearing regions of the femur.

## Methods

### Participants

Potential participants were identified via two different methods: (1) from a prospective ongoing existing study, female participants ages 50–70 were identified in our hand practice (IRB #37,532), and (2) to supplement hand clinical numbers, a retrospective anonymized chart review was performed (IRB #67,466) to identify females above the age of 18 with DXA and hand radiographs taken within one year of each other. Exclusion criteria included women with: pregnancy, inflammatory arthritis, connective tissue disease, metabolic bone disease, spondyloarthropathy, hypercalcemia, Paget’s disease, premature ovarian insufficiency, use of any bisphosphonates or denosumab in the past year or any prior thumb ligament/bone injury or surgery to the wrist or hand.

For the prospective cohort, all participants identified were for an ongoing study on carpometacarpal arthritis. Participants, both those with and without arthritis, were found in the clinic and provided their written informed consent to participate in the study. If hand or wrist x-rays were not available within the last 6 months of the participants’ enrollment, new ones were obtained. At the time of the visit, participants were asked for relevant past medical history including hand dominance, diagnosis of carpometacarpal osteoarthritis, age of menopause, thyroid issues, use of medications or supplements including Vitamin D, calcium or hormone replacement therapy, and any other pertinent medical history. Thirty-eight participants were initially identified. The study was conducted in compliance with The Declaration of Helsinki.

For the retrospective cohort, participants were identified using chart review though STAnford Research Repository (STARR) Tools. By chart review, we identified individuals with hand or wrist x-rays taken within a year of DXA scans. Patients had x-rays to evaluate for general hand or joint pain. No patients were included with fractures of the hand or wrist and all charts were screened to remove any that met the exclusion criteria. An additional 11 participants were identified by this method.

### DXAs

DXA data and methods can be viewed online at Dyrad [28]. All participants underwent a DXA scan of their hip, spine, and bilateral wrists. Participants from the prospective trial all had DXAs performed on a GE Healthcare Lunar iDXA scanner (GE Healthcare, Chicago, Illinois, USA) with enCORE software Version 16 (GE Healthcare, Chicago, Illinois, USA), retrospective chart review participants’ DXAs were taken both on a GE Lunar DXA scanner and Hologic Horizon scanner (Hologic Inc., Bedford, MA, USA) with APEX software version 5.6.0.5 (Hologic Inc., Bedford, MA, USA). BMD and T-scores were obtained for the following locations: total AP spine (L1, L2, L3, L4, L1-L4), femoral neck (left and right), total hip (left and right), 1/3 distal forearm (left and right), most distal forearm (left and right), and total forearm (left and right), forearm locations demonstrated in Supplementary Fig. 1. In one case, total AP spine was taken from L1-L3 instead of L1-L4 due to technical difficulties. Another patient did not have values from the right femur due to a prior fracture. Osteopenia was defined as T-score between − 1 and − 2.5, osteoporosis was defined as T-score of -2.5 or below. T-scores reports from the prospective group used the NHANCES/Lunar system, for the retrospective group T-scores reports were from BMDCS/Hologic, BMDCS/NHANES, NHANES/Hologic and NHANES/Lunar databases.

### Hand x-rays

**Fig. 1:**
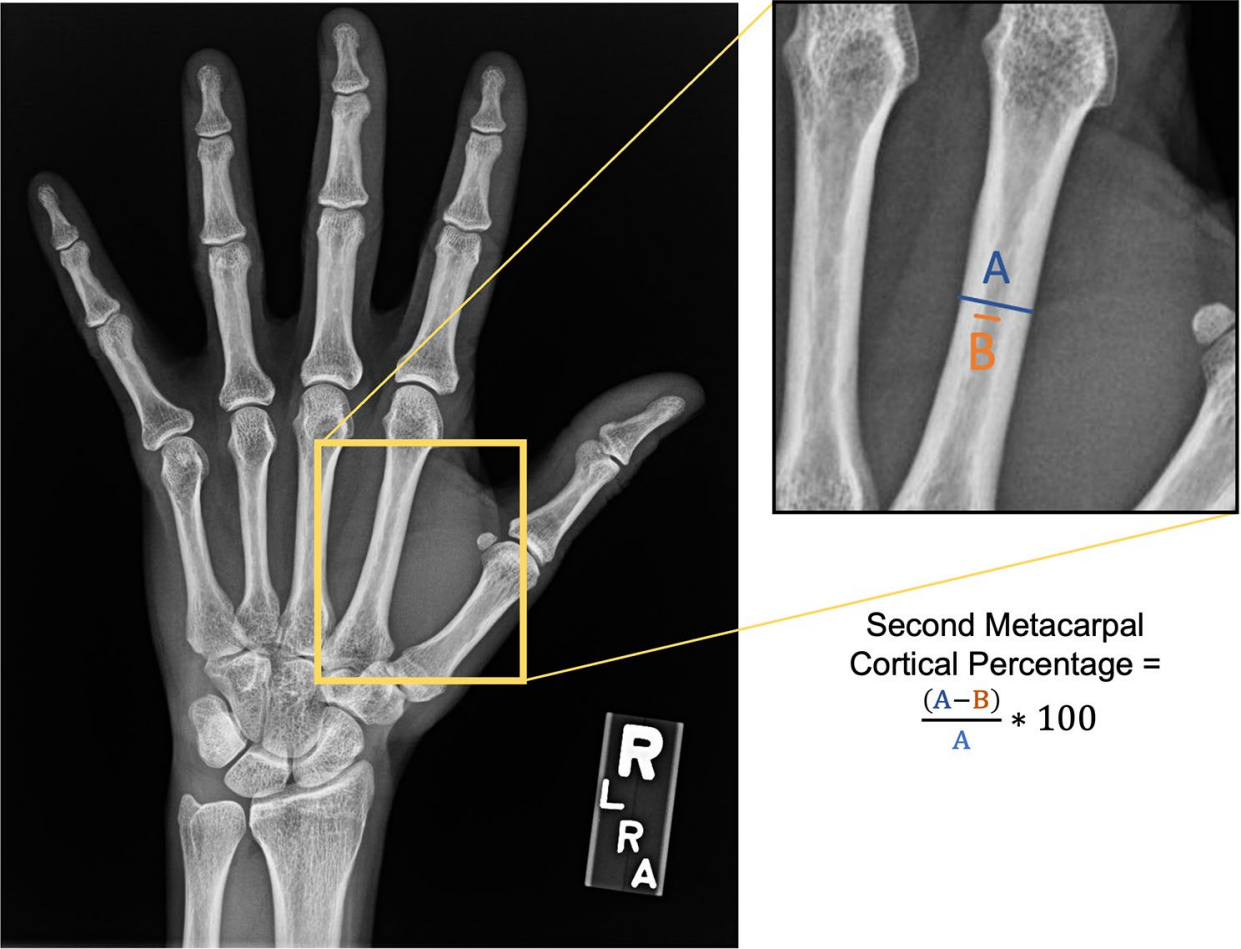
Measurement of second metacarpal percentage using hand radiographs. (**A**) measures the cortical diameter at the isthmus and (**B**) measures the intramedullary diameter

Forty-three participants with DXAs had corresponding hand or wrist x-rays within one year of each other. The PA view of the available hand or wrist x-rays was uploaded into ImageJ for image processing [31]. The mid-diaphysis of the second metacarpal was localized with the magnification function to optimize measurement. The observer chose the isthmus as the site along the second metacarpal by visually assessing the narrowest part of the cortex. The measurement tool was then used to measure the diameter of the second metacarpal at the isthmus (portion A). The second measurement was made parallel to this, at the same location, and only included the intramedullary component (portion B). We then calculated the cortical percentage by the following formula [(A-B)/A]x100 (Fig. 1) [20]. Measurements were confirmed by two independent raters and differences in calculations of the 2MCP were confirmed to be < 10% between raters. The data and methods can also be found on Dyrad [28].

### Statistical analysis

Continuous variables (2MCP, bone mineral density, and T-scores) were reported as averages with standard deviations. Both right and left measurements for forearm, femur, and hip BMD and T-scores were used when available. A paired t-test was used to determine if left and right cortical percentages significantly differed, if not and the cortical percentage from the ipsilateral side of the DXA data was missing, then missing values were imputed from the contralateral side if available. BMD was tested for normality using the Shapiro-Wilk test. BMD scores were cross calibrated between Lunar and GE systems using validated equations [29, 30]. Mixed-effects linear regression models were performed to determine the correlation between cortical percentages and BMD of the forearm, femur, hip, and spine, adjusting for age and including an adjustment for repeated measures on each side. Repeated measures ANOVA tests were used to determine differences in BMD amongst different anatomical locations, right and left sites were separated to make comparisons across all locations including the AP spine, adjusting for age. Post-hoc analysis report Bonferroni-adjusted *p*-values. Mixed-effects logistic regression models were used to determine 2MCPs between those with osteopenia and osteoporosis in the most distal and 1/3 distal forearm, adjusting for age and including adjustment for repeated measures on each side, as these are the most common sites for forearm fragility fractures. Receiver-Operator-Characteristics (ROC) were generated and areas under the curves (AUCs) were calculated to determine 2MCPs with the best specificity and sensitivity to differentiate osteopenia and osteoporosis in the most distal forearm and 1/3 distal forearm using Youden’s Index. We chose to further compare these forearm locations as they are the most common sites of forearm fragility fractures to the ROC curves generated by femoral neck T-scores. All analyses were completed in RStudio version 2021.09.1 (Boston, MA) using two-sided level of significance of 0.05.

## Results

### Participants

38 participants were enrolled in the prospective portion of this study, six were lost to follow up, leaving 32 with available DXA scans. Twenty-three of the 32 participants had corresponding hand radiographs. An additional 11 participants were added by chart review. 22 of the 43 participants had bilateral hand radiographs, 9 had only right and 6 had only left hands, for a total of 59 original 2MCP measurements. For those with left and right cortical percentages, values did not significantly differ (*p* = .15) therefore fourteen radiographs were imputed for the contralateral side when one was not available for a total of 72 cortical percentages (59 original + 14 imputed) available for analysis. All patients were female, average age of 61 (range 23–97, standard deviation 12.1), with an average age at menopause of 51.7 (range 40–62, standard deviation 4.9) more participant data can be seen in Table 1.

**Table 1:** Participant Data Including Demographics and Relevant Medical History

Age	61y (± 12.1)
Race	White: 43 (79%) Asian: 4 (9.3%) Unknown: 5 (12%)
Right Hand Dominant	36 (92%)
Age at Menopause	51.7y (± 4.9) *
BMI	25 (± 4.9)
Thyroid Issues	7 (16%)
On Hormone Replacement Therapy	3 (7%)
Carpometacarpal Osteoarthritis	18 (45%)

Quantitative values presented as average ± standard deviation. Other values are number of participants with a positive classification, in parenthesis percentage of participants

N = 43, *Three participants not included because they had not undergone menopause

### Correlation of 2MCP with BMD and T-scores & differences by osteopenia and osteoporosis

The primary hypothesis was supported in that 2MCPs were positively correlated with BMD and T-scores from all forearm locations; 2MCPs were not correlated with femoral neck, hip, or spine BMD (Table 2 and Supplementary Fig. 2a-f). Mixed effects models showed that 2MCP was significantly lower in those with osteopenia (Odds Ratio (OR) = 0.86, Confidence Interval (CI) = 0.79–0.94, *p* < .001) and osteoporosis (OR = 0.87, CI = 0.78–0.98, *p* = .019) in the 1/3 distal forearm. The 2MCP was also significantly lower in those with osteoporosis (OR = 0.82, CI = 0.73–0.92, *p* < .001) of the most distal forearm but not associated with osteopenia (OR = 0.94, CI = 0.89–1.00, *p* = .053).

**Table 2:** Correlation using Mixed Effects Models for 2MCP percentage versus BMD by anatomic location adjusted for age

	Estimate (slope)	*P*-Value
Most Distal Forearm BMD	0.003	0.001*
1/3 Forearm BMD	0.003	< 0.001*
Total Forearm BMD	0.003	< 0.001*
Femoral Neck BMD	− 0.0005	0.69
Hip Total BMD	0.001	0.23
Spine BMD	0.002	0.30

*=*P*-value < 0.05

### T-scores and BMD by anatomical location

**Fig. 2:**
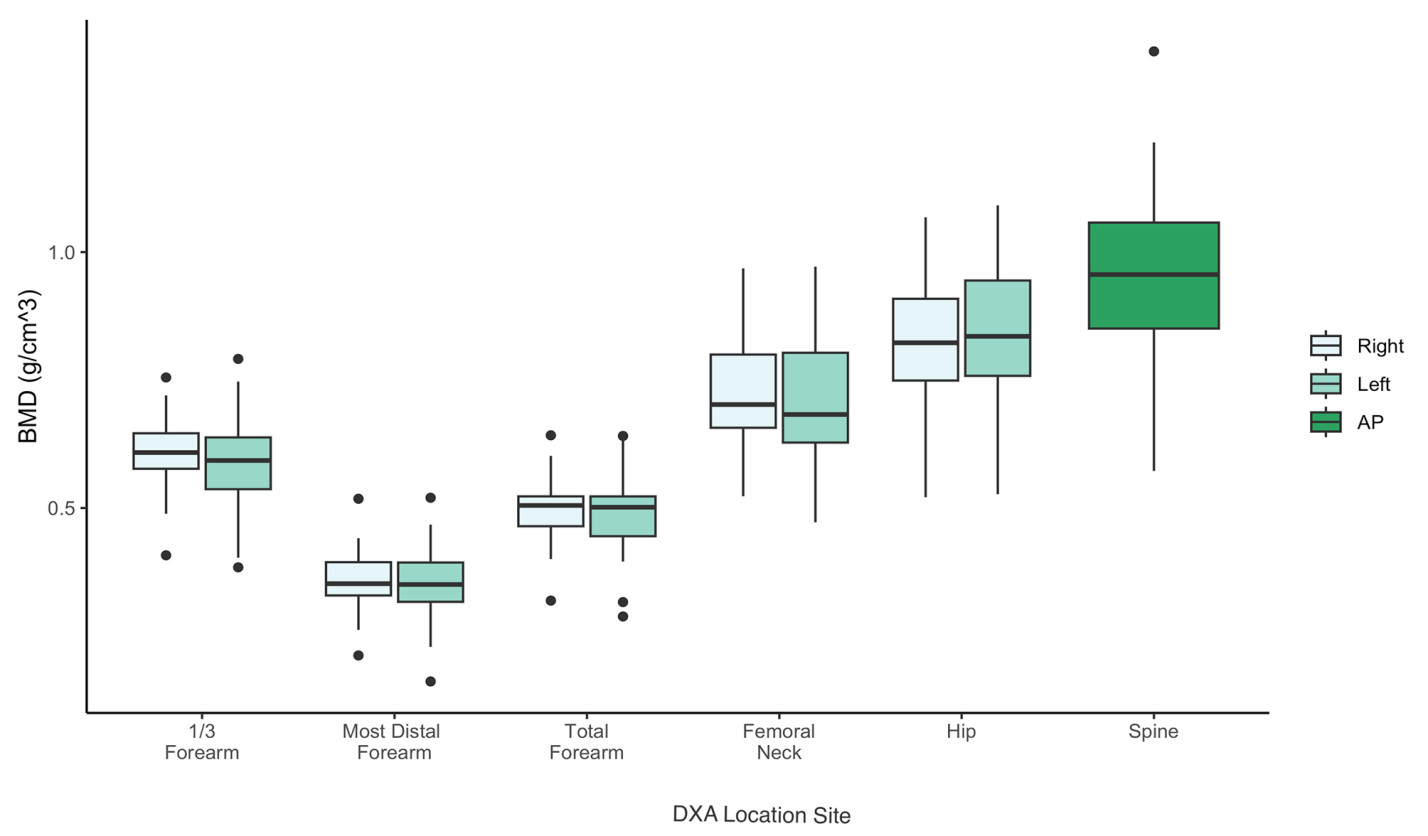
Significant differences of BMD comparisons by anatomical location. Right location is marked in light blue, left in light green, and AP in dark green. All pair-wise comparisons between forearm locations and weight bearing regions were significantly different with *p* < .001. Boxes represent interquartile range (IQR) with median bar, whiskers showing minimum to maximum, individual dots represent outliers. *P* < .001 not marked

All participants with available DXAs were analyzed. Average T-scores and BMD scores can be seen in Table 3. BMD was lowest in the most distal forearm (average 0.35 g/cm^3^ ± 0.07) and highest in the spine (0.95 ± 0.16). T-scores were lowest in the most distal forearm (-1.75 ± 1.73) and highest in the spine (-0.84 ± 1.42). A mixed-design ANOVA test revealed significant differences in BMD based on anatomical location (p<.001) (Fig. 2). Post-hoc analysis showed that BMD from all forearm locations were significantly lower than all weight-bearing regions with *P*-values < 0.001.

**Table 3:** Average BMD and T-scores across Anatomic Locations +/- Standard Deviation

	Average BMD (g/cm^3^) (± SD)	Average T-score (± SD)
Most Distal Forearm	0.36 (0.06)	-1.75 (1.73)
1/3 Forearm	0.60 (0.08)	-1.23 (1.34)
Total Forearm	0.49 (0.07)	-1.51 (1.52)
Femoral Neck	0.71 (0.11)	-1.16 (0.94)
Hip	0.83 (0.13)	-0.83 (1.02)
Spine	0.95 (0.16)	-0.84 (1.42)

### 2MCP cutoff values determine osteopenia and osteoporosis in the forearm

**Fig. 3:**
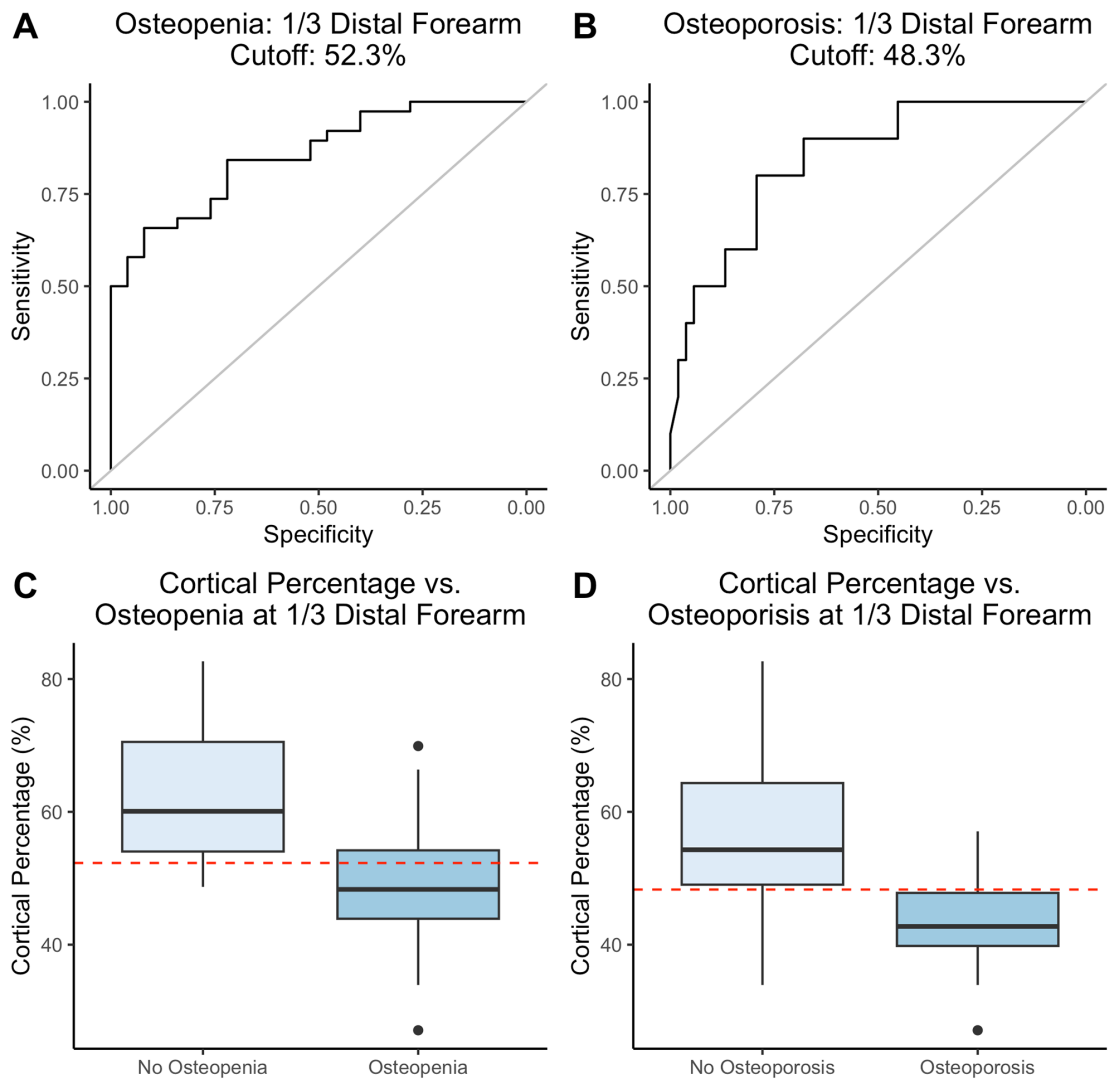
Optimal cutoff value of cortical percentage for 1/3 Distal Forearm. Receiver Operator Curves for cortical percentage cutoff for optimal sensitivity and specificity for osteopenia (**a**) and osteoporosis (**b**) of 1/3 distal forearm, box and whiskers blot showing cutoff (dashed red line) of 52.3% for osteopenia (**c**), and 48.3% for osteoporosis (**d**) of the 1/3 distal forearm

2MCPs were compared with the osteopenia and osteoporosis categorizations in the most distal and 1/3 distal forearm. We generated ROC curves to determine cortical percentages that were indicative of corresponding osteopenia and osteoporosis. The optimal 2MCP for determining osteopenia of the 1/3 distal forearm was 52.3%; this percentage correctly identified 65.8% of participants with osteopenia of the 1/3 forearm (sensitivity 65.8%) and correctly ruled out 92% of participants without (specificity 95%) with an AUC of 0.86 (Fig. 3a&c). The optimal 2MCP for detecting osteoporosis of the 1/3 forearm was 48.3% with a sensitivity of 80% and specificity of 79.2% and an AUC of 0.85 (Fig. 3b&d).

**Fig. 4:**
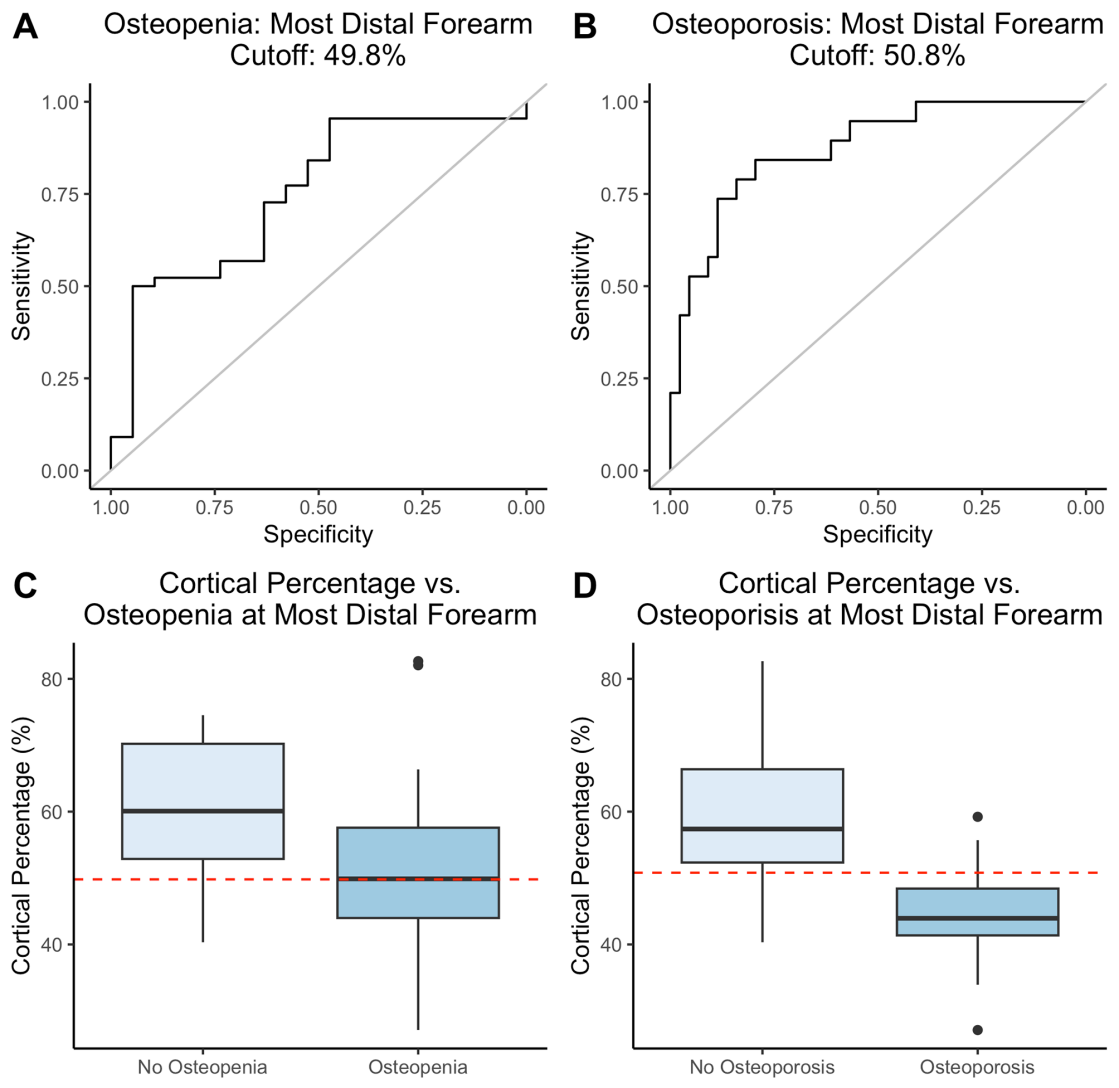
Optimal cutoff value of cortical percentage for Most Distal Forearm. Receiver Operator Curves for cortical percentage cutoff for optimal sensitivity and specificity for osteopenia (**a**) and osteoporosis (**b**) of the most distal forearm, box and whiskers blot showing cutoff (dashed red line) of 49.8% for osteopenia (**c**), and 50.8% for osteoporosis (**d**) of most distal forearm

The optimal 2MCP to determine osteopenia of the most distal forearm was 49.8% which had a sensitivity of 50% and specificity of 94.7% and AUC of 0.75 (Fig. 4a&c). A 2MCP cutoff of 50.8% was optimal for determining osteoporosis of the most distal forearm with sensitivity of 84.2% and specificity of 79.5% and AUC of 0.87 (Fig. 4b&d).

In comparison with the ROC generated from femoral neck T-scores (used in FRAX scores), the predetermined cutoff of < -1.0 for femoral neck T-score osteopenia correctly predicts osteopenia of the 1/3 distal forearm in 82% of participants (sensitivity 82%) and correctly rules out osteopenia in 77% of participants (specificity 77%) with an AUC of 0.86. The femoral neck T-score of -2.5 used for osteoporosis has a sensitivity of 17% and specificity of 95% for predicting osteoporosis of the 1/3 distal forearm, AUC of 0.88. In the most distal forearm, the femoral neck T-score of -1.0 had a sensitivity of 73%, specificity of 71% and AUC of 0.82 for predicting osteopenia. The femoral neck T-score of -2.5 had a sensitivity of 10%, specificity of 96% and AUC of 0.79 for predicting osteoporosis of the most distal forearm.

## Discussion

In this study, we confirmed our hypothesis that cortical percentage is most strongly associated with forearm BMD, that cortical percentage can accurately differentiate forearm osteopenia and osteoporosis, and that 2MCP is a superior screening determinant of osteoporosis in the forearm than the femoral neck.

Similar to our study, previous research has demonstrated BMD from weight-bearing regions overestimates forearm BMD. However, weightbearing sites are used for fracture risk assessment, which may provide patients and healthcare teams with an inaccurate picture of the true risk of forearm fractures [15]. Our findings from the ROCs support that 2MCP is more sensitive than femoral neck T-scores for predicting forearm osteoporosis. This suggests that using femoral neck T-scores to detect forearm osteoporosis would result in many false negatives.

Using 2MCP would allow clinicians to rule-in the possibility of osteoporosis for patients who may otherwise go undiagnosed. We recommend adopting the optimal 2MCP cutoff of 48.3% to predict osteoporosis of the 1/3 distal forearm or 50.8% to predict osteoporosis of the most distal forearm. Both of these values provide higher sensitivity (1/3 distal: 2MCP 80% vs. femoral neck 17%; most distal: 2MCP 84% vs. femoral neck 10%) to prevent false negatives which is ideal for screening tests [32].

In addition, we found BMD from all forearm locations was significantly lower than femoral neck, hip, and spine BMD. Previous studies confirm these findings that the forearm BMD is not accurately represented by weightbearing bones. Miyamura and colleagues evaluated postmenopausal women with and without a history of distal radius fractures in a case-controlled study. Patients with fractures had significantly lower BMD values calculated from DXA scans of the radius but no differences in femoral neck or spine BMD compared to patients who had not sustained a fracture of the radius [14]. Additionally, both femoral neck and spine T-scores overestimated T-scores in the forearm of the fracture group [14]. Ma and colleagues further demonstrated the importance of local forearm BMD assessment with retrospective data showing patients who sustained distal radius fractures had significantly lower forearm BMD and low T-scores about the radius were a predictive risk factor for fracture, while femoral neck, hip and spine T-scores were not significant risk factors for the occurrence of distal radius fractures [27]. These studies highlight the importance of determining local forearm osteopenia and osteoporosis separate from weight-bearing locations.

In another study, Rozental and colleagues compared bone architecture using high-resolution peripheral quantitative computed tomography between premenopausal women with and without distal radius fractures [33]. They detected differences in osseous architecture, trabecular thickness, density, and separation of the forearm in the fracture group but no differences in spine or femoral neck BMD between the groups [33]. An additional study of elderly patients with cardiovascular disease found that using distal radius BMD in the diagnosis of osteoporosis had a significantly superior diagnostic sensitivity than lumbar spine BMD (*p* < .0001), which raises concerns for the clinical utility lumbar spine and femoral neck BMD have in screening for this population [34]. Similar to our findings, these studies indicate that femoral neck and spine BMD scores overestimate bone quality in the forearm leaving patients and healthcare providers unaware of forearm fragility. These findings and our study corroborate the potential utility of the 2MCP for predicting forearm BMD as a tool to initiate earlier intervention for those who may benefit from activity modification, dietary supplementation, and pharmacologic intervention.

While we found that the 2MCP correlated with total forearm density, we did not see a correlation with hip, femoral neck or spine density or T-scores. This differs from a few previous studies on 2MCPs that correlated with hip BMD and T-scores [20, 24]. Differences could be due to the larger range of hip T-scores (-4.9 to 2.3 and − 4.1 to 2.3) in these studies, likely due to the inclusion of males or larger populations. Although we did not see a correlation of 2MCP with weight-bearing regions this does not imply that future relationships will not exist if bone loss progresses. In fact, we could be detecting early alterations in BMD at the forearm with 2MCPs.

Limitations of our study include single-sex participants, a relatively small sample size, and the use of two different methods (a prospective and retrospective design) to obtain data, which restrict the generalizability of our findings. Although only women were included in this study, the vast majority of distal radius fractures occur in women, substantiating its clinical use [35]. In addition, the current sample size did not provide adjustment for variables such as BMI, thyroid disorders, if patients were taking Vitamin D or Calcium, or previously used hormone replacements therapy or bisphosphonates. While the sample size gave us the power to detect a large correlation (effect size = 0.05) between BMD and cortical percentage, we were underpowered to detect an effect this large for some anatomical locations (primarily locations other than the forearm).

Limitations due to the prospective and retrospective nature of our study include that DXA data was obtained off various systems. While this may introduce error, improvements have been made such that correlations of measurements between manufacturers is strong [36]. Additionally, although different reference databases were used for T-scores, it has been shown that absolute BMD can provide a robust analysis, therefore the use of BMD was included. While T-scores were used for ROC analysis, the data was based off of categorization (osteopenia and osteoporosis), which has been shown to coincide between databases [37]. We recognize the generalizability of this study must be evaluated at different institutions prior to initiation into clinical practice and future studies are needed to determine if 2MCP can be used as a clinical measurement taken over time to assess risk of low BMD and fracture of the forearm.

In summary, our study demonstrates that opportunistic hand x-rays can serve as an assessment tool for patients at risk of developing osteopenia and osteoporosis of the forearm. It is known that wrist fractures are the second most common fracture type in those older than 50, after vertebral fractures, and are associated with significant morbidity and mortality [3, 6, 10]. Our study demonstrated cortical percentages from hand radiographs can accurately predict low BMD in the forearm with high sensitivity and specificity, and that many of these cases would have been overlooked by FRAX estimates due to higher BMD in the femoral neck. This implies that screening hand x-rays in women are superior to current assessment tools for predicting fracture risk. Expansion of screening to hand x-rays would enable early detection and treatment with a low-cost, low-morbidity alternative to an additional DXA scan. Ultimately early screening proposes to reduce the rate of future fragility fractures and help offset the $17 billion, and growing, economic burden associated with osteoporosis [6, 38].

## Conclusions

This study demonstrates that the 2MCP is strongly correlated with forearm BMD. This x-ray measurement is both an accurate marker for detecting osteoporosis of the forearm and more sensitive than femoral neck T-scores at detecting early BMD changes. These findings support the expansion of osteoporosis screening to the lower-cost PA hand x-ray. Future investigation will focus on confirming the optimal 2MCP cutoff for screening for osteopenia and osteoporosis at scale. We strive for integrating automatic 2MCP measurements into existing PAC systems to optimize clinical implementation on a universal scale.

### Electronic supplementary material

Below is the link to the electronic supplementary material.

Supplementary Material 1

Supplementary Material 2

## Data Availability

Data are made accessible via Dyrad’s website, doi:10.5061/dryad.6hdr7sr57.
