# Supplementary figures and images for: Opportunistic hand radiographs to screen for low forearm bone mineral density: a prospective and retrospective cohort study
Source: BMC Musculoskelet Disord. 2024 Feb 20;25:159. doi: 10.1186/s12891-023-07127-w (PMC10877789; doi:10.1186/s12891-023-07127-w)

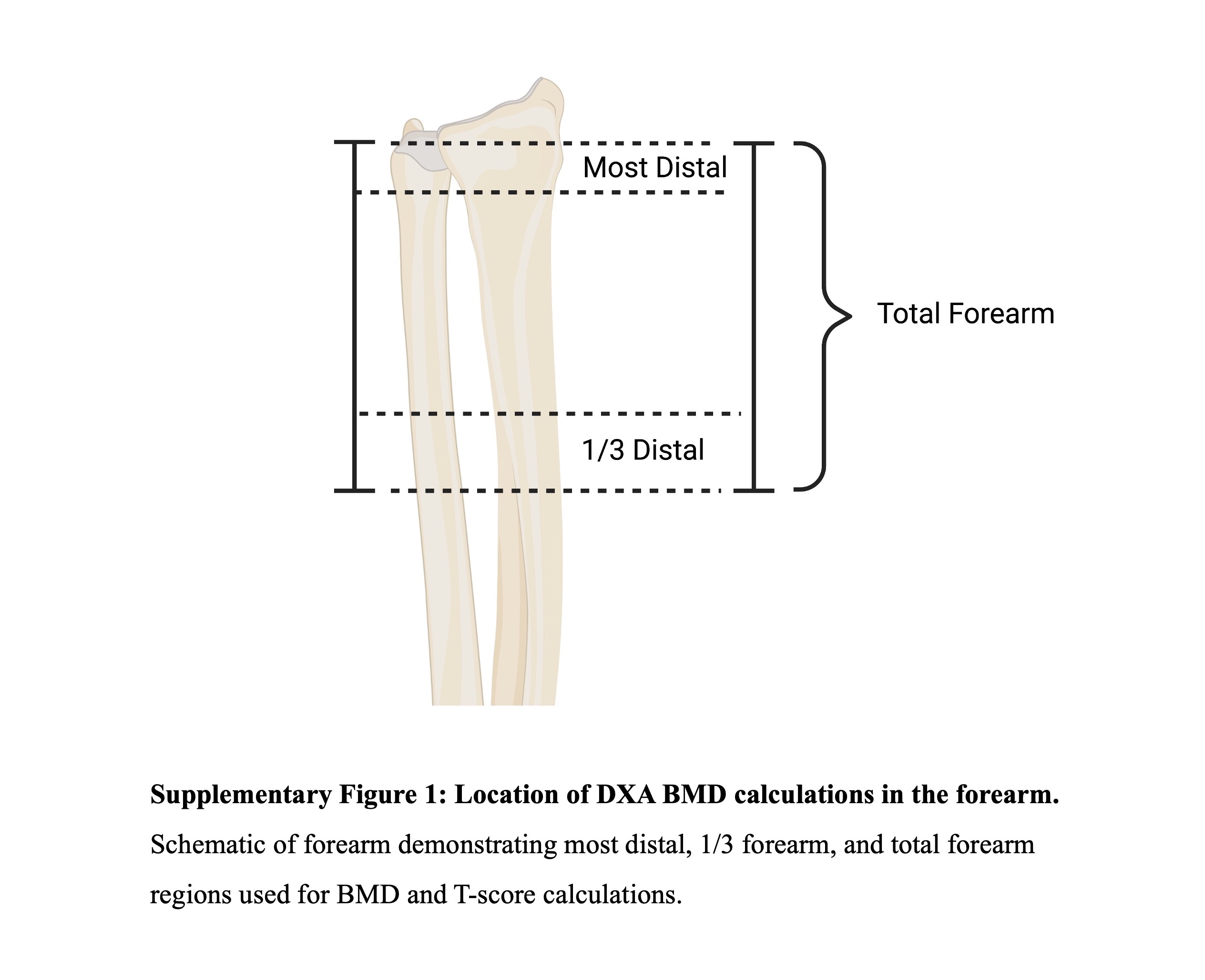

Supplement: Supplementary file 1 — Supplementary Material 1 [file 12891_2023_7127_MOESM1_ESM.jpg]
