# Supplementary material for: Opportunistic hand radiographs to screen for low forearm bone mineral density: a prospective and retrospective cohort study
Source: BMC Musculoskelet Disord. 2024 Feb 20;25:159. doi: 10.1186/s12891-023-07127-w (PMC10877789; doi:10.1186/s12891-023-07127-w)
Supplement: Supplementary file 2 — Supplementary Material 2 [file 12891_2023_7127_MOESM2_ESM.pdf]

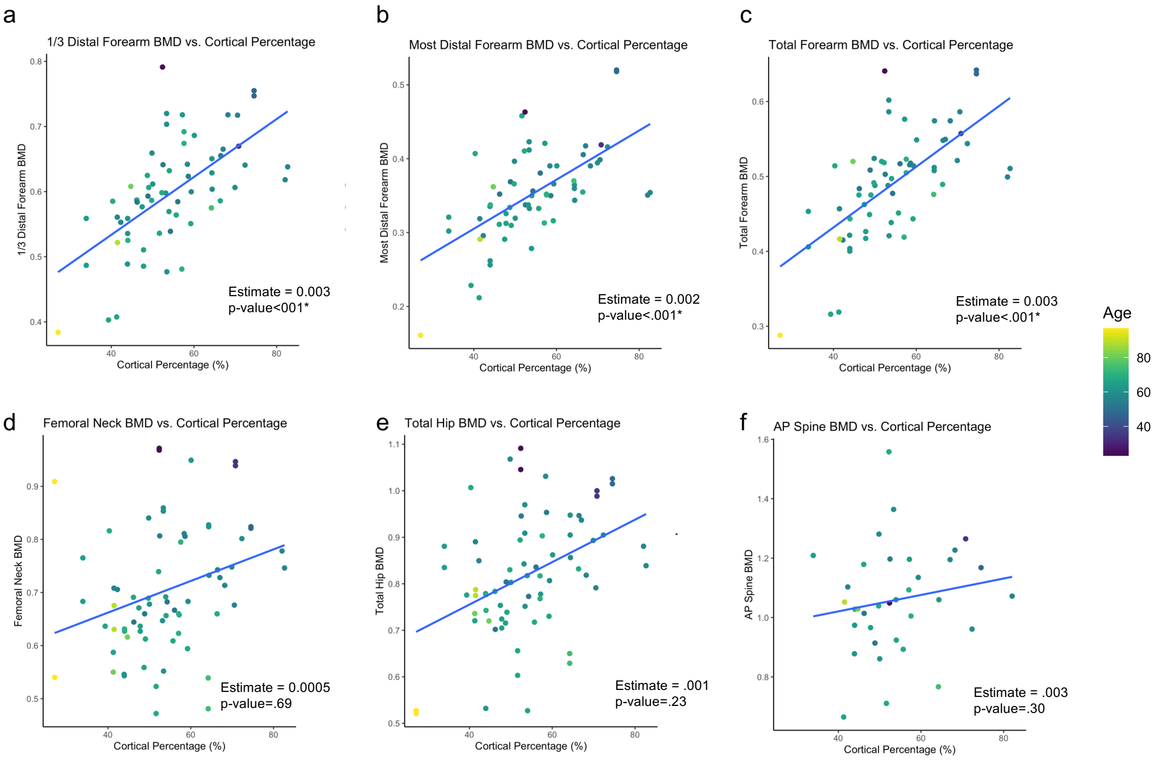

Supplementary Figure 2: Correlations of Second Metacarpal Cortical Percentage vs BMD at various anatomic locations. Graphs represent mixed effects models with age-adjusted results for 1/3 distal forearm (a), most distal forearm (b), total forearm (c), femoral neck (d), hip (e), and spine (f). Bar on right represents age scale by color. Estimates represent slope for every 1% change in BMD.
* indicates p-value <.05.
